# Comparison of the functional features of the pump organs of *Anopheles sinensis* and *Aedes togoi*

**DOI:** 10.1038/srep15148

**Published:** 2015-10-14

**Authors:** Young-Ran Ha, Seung-Chul Lee, Seung-Jun Seo, Jeongeun Ryu, Dong-Kyu Lee, Sang-Joon Lee

**Affiliations:** 1 Division of Integrative Bioscience and Bioengineering, Pohang University of Science and Technology, Pohang, 790-784, Republic of Korea; 2 Center for Biofluid and Biomimic Research, Pohang University of Science and Technology, Pohang, 790-784, Republic of Korea; 3 Pohang Accelerator Laboratory, Pohang University of Science and Technology, Pohang, 790-784, Republic of Korea; 4 Department of Mechanical Engineering, Pohang University of Science and Technology, Pohang, 790-784, Republic of Korea; 5 Department of Biology, Kosin University, Busan 516-36, Republic of Korea

## Abstract

Mosquitoes act as vectors for severe tropical diseases. Mosquito-borne diseases are affected by various factors such as environmental conditions, host body susceptibility, and mosquito feeding behavior. Among these factors, feeding behavior is affected by the feeding pump system located inside the mosquito head and also depends on the species of mosquito. Therefore, the 3D morphological structures of the feeding pumps of *Aedes togoi* and *Anopheles sinensis* were comparatively investigated using synchrotron X-ray microscopic computed tomography. In addition, the feeding behaviors of their pumping organs were also investigated using a 2D X-ray micro-imaging technique. *An. sinensis*, a malarial vector mosquito, had a larger feeding pump volume than *Ae. togoi* in the static or resting position. Interestingly, the two species of mosquitoes exhibited different feeding behaviors. *Ae. togoi* had a higher feeding frequency and expansion ratio than *An. sinensis*. *Ae. togoi* also exhibited F-actin localization more clearly. These distinctive variations in feeding volumes and behaviors provide essential insight into the blood-feeding mechanisms of female mosquitoes as vectors for tropical diseases.

Mosquitoes are vectors of many diseases, including malaria, dengue, Japanese encephalitis, yellow fever, and filariasis^1^. These vector-borne diseases are major global public health threats^2^ and pose complex biological problems, such as the emergence of drug-resistant strains of pathogens and insecticide-resistant vector populations^1^. Therefore, medical research teams have been struggling for many years to develop effective vaccines or drugs for mosquito-borne diseases^3^.

Pathogens in mosquitoes are always transmitted to the host during probing or blood feeding^4^. The pump organs positioned in the head create the suction pressure necessary to suck blood through the blood canal of its proboscis^5^. As a result, liquid-phase fluids are sucked through a tightly sealed proboscis^6^. Most blood-feeding arthropods have piercing-sucking mouthparts at their prosoma to feed on blood^5^. However, the mouthparts of blood-feeding arthropods have different morphological structures and functional mechanisms^5^. Mosquitoes regulate blood feeding by controlling the two pump organs the cibarial dilator pump (CP) and the pharyngeal dilator pump (PP)^7^. The valve-like organ (Vap) positioned between the two pumps appears to prevent backflow in the conduit connecting the two pumps (C-P)^7^. The two pump organs assist in the feeding of liquid food and in the effective regulation of the blood-sucking process. The CP located at the curved conduit takes up blood from the host through the food canal. The curved conduit is directly connected to the proboscis and to the digestive organs in the main body. Without the curved conduit in the food passage, it would be difficult for mosquitoes to hunt and pierce the skin of a host to suck its blood^8^. The morphological and functional features of the pump system of a female mosquito have a strong influence on its feeding behavior, thus affecting host exposure to pathogens^9^.

Mosquitoes exhibit different postures in the static or resting condition. Interestingly, *Anopheles* and *Aedes* show different sitting postures during the resting and feeding phases. *Anopheles* commonly rests by thrusting its abdomen upward to straighten the proboscis and main body, whereas *Aedes* lies parallel to its resting surface. These two mosquitoes spread different types of diseases. The transmission of malarial parasites to humans usually occurs through *Anopheles*, whereas *Aedes* transmits dengue and yellow fever^10^. The sitting posture of mosquitoes was suggested to reflect the kinematic characteristics of their structures and behaviors^11^.

In this study, the 3D morphological features of the pumping organs of *An. sinensis* and *Ae. togoi* were investigated using synchrotron X-ray microscopic computed tomography (SR-μCT). To evaluate the blood feeding volume of a female mosquito, the two pump chambers were extracted from its 3D reconstructed image. The volumetric variations and feeding behaviors of the feeding pump chambers of *An. sinensis* and *Ae. togoi* were quantitatively compared. In addition, differences in the actin-based structures of the heads of *An. sinensis* and *Ae. togoi* were investigated. These experimental results will aid in understanding the biophysical characteristics of the pumping organs of *An. sinensis* and *Ae. togoi*, which transmit different tropical diseases.

## Results

### SEM images of the female mosquito heads

**Figure 1:**
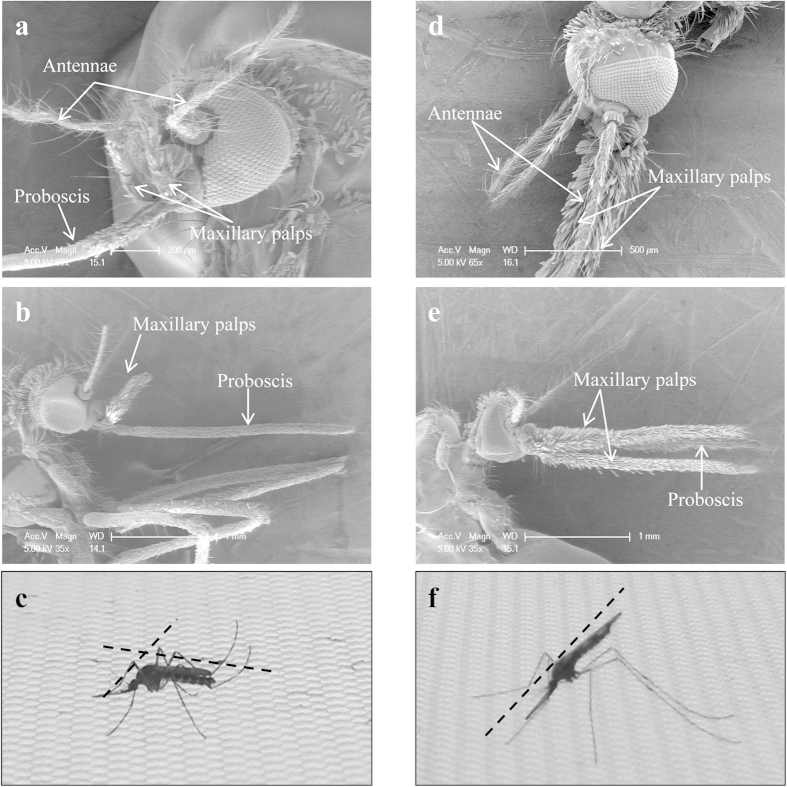
SEM images of the heads and proboscises of *Ae. togoi* (**a,b**) and *An. sinensis* (**d,e**) female mosquitoes. Images of the resting state of *Ae. togoi* (**c**) and *An. sinensis* (**f**).

Figure 1a–d show typical SEM images of the proboscis and head parts of *Ae. togoi* and *An. sinensis*. The length of the maxillary palp of *Ae. togoi* is shorter than that of *An. sinensis* (Fig. 1b,e). Figure 1c,f show images of the typical sitting postures of the two different species of mosquitoes in the static or resting state.

### 3D reconstruction of the mosquito heads

The feeding pump organs are in the head of a mosquito. The CP is located under the clypeus, and the PP is located above and behind the cibarial pump. The CP conduit is located between these two pumps^12^. The CP has a curved shape and is supported against the pull of dilator muscles positioned between the sub-esophageal ganglion and the brain^12^.

**Figure 2:**
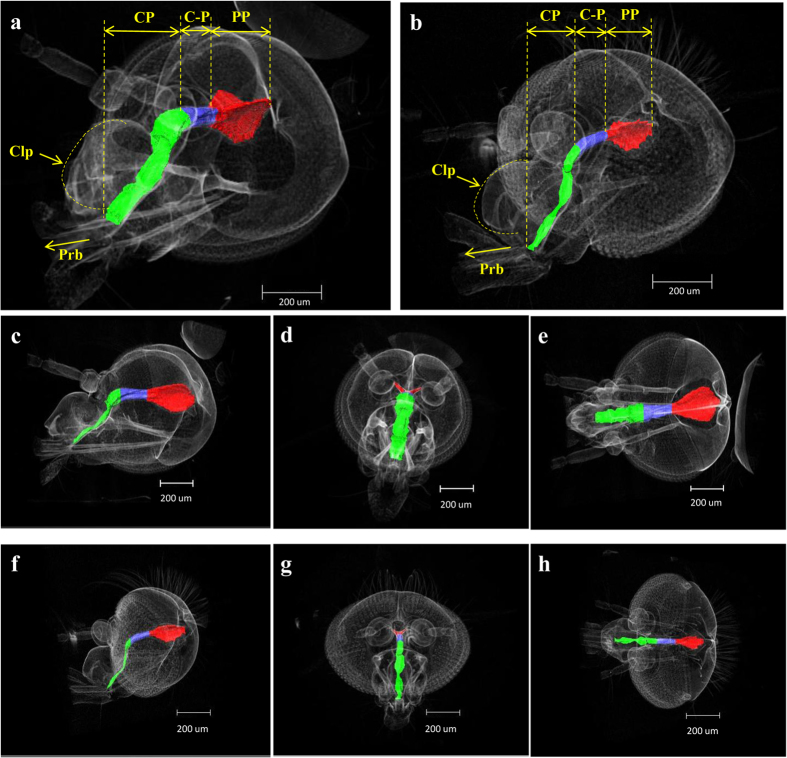
3D morphological structures of the heads of *An. sinensis* (**a**) and *Ae. togoi* (**b**) female mosquitoes reconstructed using SR-μCT. The viewing angle is slightly turned from the coronal view. (**c**) Sagittal view, (**d**) coronal view, (**e**) axial view of *An. sinensis*. (**f**) Sagittal view, (**g**) coronal view, (**h**) axial view of *Ae. togoi*.

The sagittal, coronal, and axial images of the feeding pumps are shown in Fig. 2. Using an image-segmentation tool, the feeding pumps of the mosquitoes were quantitatively analyzed, and the pixels in the salient image regions were clustered. Mosquito samples fixed with an ethanol solution were selected to represent the reference cases of the static state. Based on the reference images, the pump systems of these two different species of mosquitoes were found to be clearly different.

### SR-μCT cross-sectional image of the mosquito feeding pump organs

**Figure 3:**
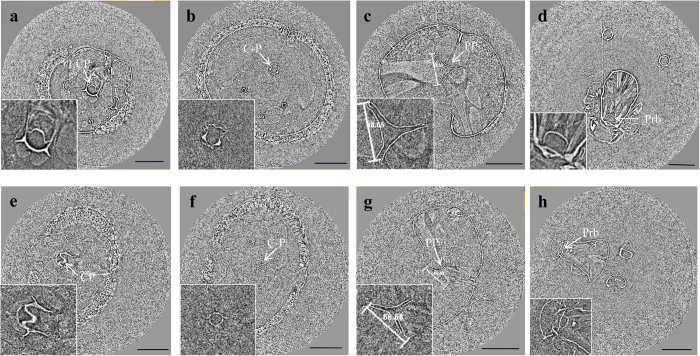
Cross-sectional images (**a–h**) of the pump systems reconstructed using SR-μCT. The Cross-sectional shape and area of the pump organs are changed according to the sectional position. SR-μCT cross-sectional images of *An. sinensis* (**a–d**) and *Ae. togoi* (**e–h**). (**a,e**) Cross-sectional images of the CP. (**b,f**) Cross-sectional images of the C-P. (**c,g**) Cross-sectional images of the PP. (**d,h**) Cross-sectional images of the proboscis.

To compare the pump systems of the two mosquitoes, their cross-sectional images are shown in Fig. 3. Figure 3a,e show the dorsal inner walls of the CP. This CP is located near the cibarial dome situated directly above the cibarial armature^12^. The cross-sectional images of the PP are shown in Fig. 3c,g. The PP has a triangular shape comprising three sclerotized plates. These three plates, one dorsal plate and two ventro-lateral plates, are drawn together during the contraction process of the dilator muscles. These contraction processes induce the formation of the posterior pharyngeal valve^12^.

The pumps of *Ae. togoi* and *An. sinensis* have similar shapes as those obtained in the static state. The size of the sclerotized plates of the PP in *An. sinensis* is mostly larger than that of *Ae. togoi* (Fig. 3). For example, the distance between the two points of the sclerotized plates of *An. sinensis* and *Ae. togoi* is 319.12 ± 105.70 μm and 235.31 ± 71.63 μm, respectively (Fig. 3c,g).

### Volume variation of the pumps in the static and expansion states

The blood-feeding pump organs were segmented from the 3D reconstructed head of the two different types of female mosquitoes to measure variations in volume. To estimate the pump volume, the volumes of the feeding pump organs of 4 to 5 similarly sized mosquito samples were statistically averaged.

**Figure 4:**
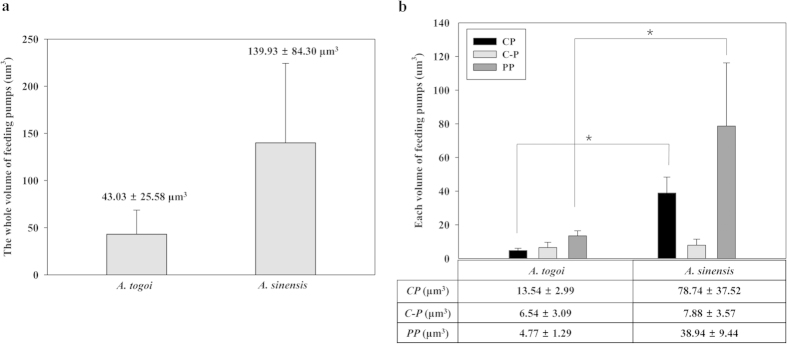
Comparison of the whole volumes of the blood-feeding pumps at rest in *An. sinensis* and *Ae. togoi*. (**a**) The whole volume of the CP, C-P, and PP of both mosquitoes. (**b**) A volumetric comparison of each pump organ for both mosquitoes. Error bars indicate standard deviation; *P < 0.05.

The whole volumes of the feeding pumps organs in the static state are compared in Fig. 4a. The volumes of the CP, C-P, and PP of both mosquitoes are compared in Fig. 4b. The whole volumes of the feeding pumps of *Ae. togoi* and *An. sinensis* are 43.03 ± 25.25 μm^3^ and 139.93 ± 84.30 μm^3^, respectively (Fig. 4a). The whole volume of *An. sinensis* is much larger than that of *Ae. togoi* in the static state. The volumes of the CP are approximately 4.77 ± 1.29 μm^3^ and 38.94 ± 9.45 μm^3^ for *Ae. togoi* and *An. sinensis*, respectively. The volumes of the PP are 13.54 ± 2.99 μm^3^ and 78.74 ± 37.52 μm^3^ for *Ae. togoi* and *An. sinensis*, respectively. The volumes of the CP and PP are larger in *An. sinensis* compared with those in *Ae. togoi*. The volumes of the C-P in both mosquito species were comparable (Fig. 4b).

**Figure 5:**
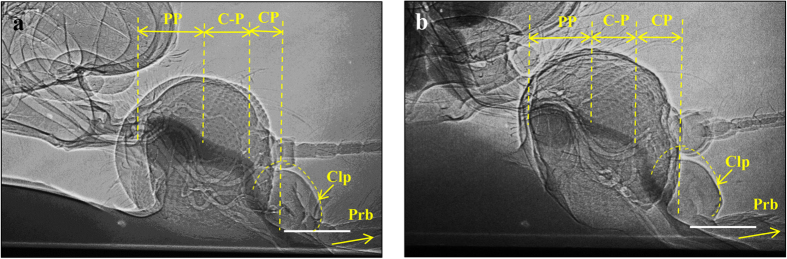
2D X-ray images showing the sagittal view of the head of a mosquito sucking a dilute iodine solution. The expansion state of the CP, C-P, and PP of *An. sinensis* (**a**) and of *Ae. togoi* (**b**). Scale bar indicates 200 μm.

Figure 5 shows a sagittal view of the head of a mosquito sucking a diluted iodine solution. The whole volume of the feeding pumps does not show a large difference in the expansion process. The C-P of *An. sinensis* is more expanded compared with that of *Ae. togoi* (Fig. 5).

### Systematic operation of the two pumping organs in both mosquitoes

**Figure 6:**
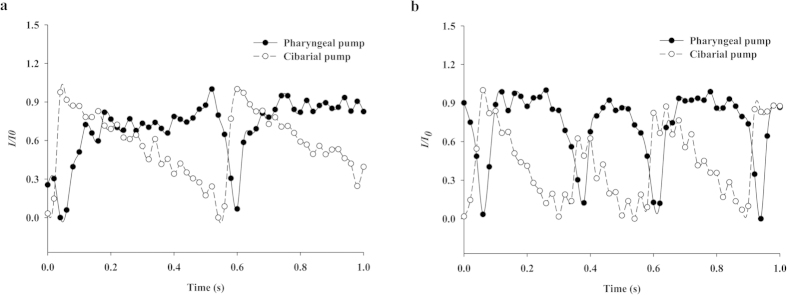
Temporal variations of the two pump organs of *An. sinensis* (**a**) and *Ae. togoi* (**b**). Each data point represents the mean value. The values are the means ± standard deviations.

Figure 6 shows temporal variations in the instantaneous volumes of the two pumps, the CP and the PP, of the two mosquitoes. The fluid-sucking process of a female mosquito occurs as follows: The mosquito sucks the diluted iodine solution. The CP begins to expand to suck the liquid food, whereas the PP remains static. The light intensity of the CP region gradually increases as the iodine solution accumulates. Simultaneously, the PP starts to expand with a certain time interval. Following the contraction and expansion processes of the two pumps, the mosquito completes one feeding cycle^13^.

The feeding cycles of both mosquitoes are less than one second. To analyze the feeding cycles quantitatively, the intensity values of the test samples were condition-averaged by assigning the instant of the maximum intensity value in each period as the reference time (*t* = *0*). High-intensity values correspond to the expansion state of the pumping organ, in which the penetration depth of the X-ray beam is increased. During the pumping process, the two pump organs exhibit a phase shift in the expansion phase. The presence of the phase shift indicates that the temporal volume variations of the two pumps are dynamically coordinated and synchronized in each feeding cycle^8^. The light intensity of the CP starts to increase before the PP begins to rise. Thus, the feeding cycles of the two different mosquitoes are clearly distinguished. The feeding frequency of *An. sinensis* is 2.0 ± 1.2 Hz and that of *Ae. togoi* is 3.9 ± 1.2 Hz.

**Figure 7:**
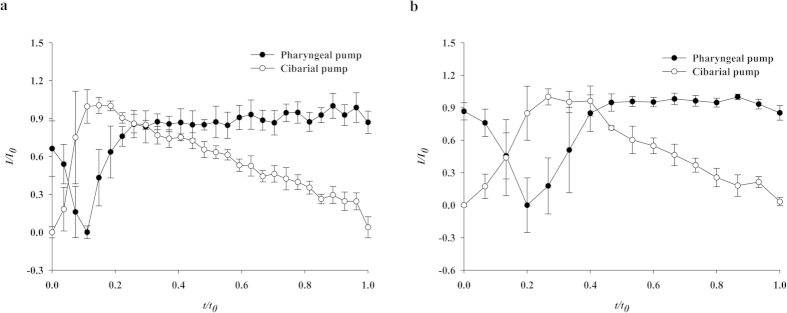
Cyclic volume variations of the two pumps, CP and PP, of. *An. sinensis* (**a**) and *Ae. togoi* (**b**). The values are the means ± standard error of the mean (SEM). Intensity (*I*) is normalized by the initial intensity *I*_*0*_. *t/t*_*0*_ is the dimensionless time.

Figure 7 represents cyclic volume variations of the two pumps of a typical *An. sinensis* female mosquito (a) and a typical *Ae. togoi* female mosquito (b). The experimental data were obtained by phase-averaging five feeding cycles for each species. The expansion periods of the CP and PP of *Ae. togoi* in a feeding cycle are longer than those of *An. sinensis*. The expansion ratio of CP/PP for *Ae. togoi* is 1.6-fold greater than that of *An. sinensis*. These results demonstrate that the two mosquito species exhibit markedly different pumping behaviors in their pumping frequencies and expansion ratios.

### Cytochemistry of actin of both mosquitoes

**Figure 8:**
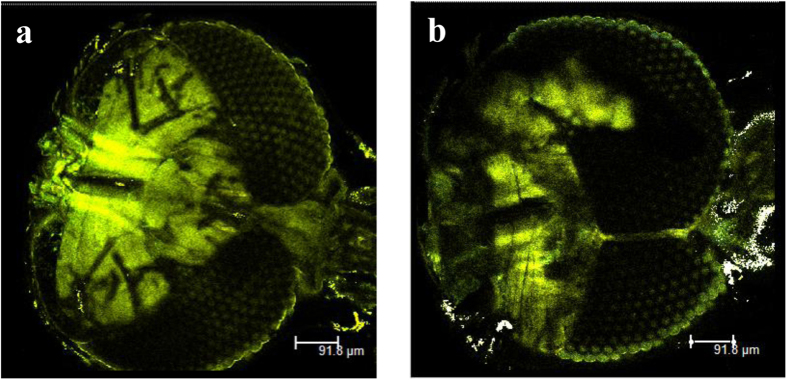
TPM images of the mosquito heads. Images of *Ae. togoi* stained with phalloidin-FITC (**a**). Images of *An. sinensis* stained with phalloidin-FITC (**b**).

To investigate the local distribution of actin inside the heads, the mosquitoes were dissected and stained with FITC-conjugated phalloidin, which specifically binds to fibrous actin (F-actin). By using the TPM system, the local distribution of actin associated with muscle actuations was captured. As shown in Fig. 8, the two species of mosquitoes exhibit F-actin expression. Near the pump chamber PP, F-actin is clearly more localized in *Ae. togoi* than in *An. sinensis*. The intensity of F-actin is 44.3% higher in *An. sinensis* compared with *Ae. togoi*. The local distribution of actin inside of midgut, please refer to the supplementary information paper which accompanies this paper.

## Discussion

A female mosquito sucks blood to provide protein to her eggs. During this blood-feeding process, mosquitoes can transmit pathogens or parasites to the host. To feed on blood from the host, female mosquitoes utilize the pumping organs located in their heads^8^. The pumping organs in their heads create a large negative suction pressure to suck the blood through the proboscis into their mouths^5^. A few theoretical studies have been conducted on the blood-feeding process of mosquitoes. However, these studies have focused only on the pumping characteristics of a single mosquito species. In this study, we compared the blood-feeding pump systems of *An. sinensis* and *Ae. togoi*. Understanding the pump system of two different species of mosquitoes may help to understand differences in feeding behavior.

In this study, we compared the sizes of the pharynx pumps in the two different mosquitoes. The cross-sectional area of the *An. sinensis* PP is larger than that of *Ae. togoi* at rest. However, the volumes of the C-P in the expansion state are not appreciably different between the two species of female mosquitoes. Interestingly, *An. sinensis* and *Ae. togoi* also exhibit different sitting postures during rest or feeding. This feeding posture may affect the expansion or contraction of the feeding pumps. *An. sinensis* may have more pressure in its pumps compared with *Ae. togoi*. Therefore, at rest, *An. sinensis* may not contract its feeding pumps completely.

The differences between the feeding behaviors of the two mosquitoes are not fully understood yet. Therefore, in this study, we explored the distinctive feeding behaviors of the two mosquitoes. The expansion period of the pump organs of *Ae. togoi* in one feeding period is longer compared with that of *An. sinensis*. In addition, the pumping frequency of *Ae. togoi* is faster than that of *An. sinensis*; although *Ae. togoi* has a longer expansion period, it has a faster pumping frequency. This result implies that *Ae. togoi* contracts its feeding pump much faster than *An. sinensis*. This phenomenon is closely related to the different volumes of the feeding pumps of the two mosquitoes. The volume of the feeding pumps of *An. sinensis* is much larger than that of *Ae. togoi* at rest. However, the pump volume in the expansion state is not noticeably different in either mosquito species. This result may explain why *Ae. togoi* needs a longer expansion period compared with *An. sinensis*. *Ae. togoi* has a much smaller pump volume at rest compared with *An. sinensis*. Therefore, *Ae. togoi* needs more time to fill its pump chamber with the fluid. These results suggest some explanations for the observed differences in feeding behaviors between the two mosquito species. First, different sitting postures may explain the distinct mechanistic feeding behaviors of the two mosquitoes. The feeding posture appears to be closely related to the pumping pressure, feeding rate, salivary flow, and transmission of parasitic diseases. A previous study showed that in mosquitoes that suck blood via capillary feeding, blood is driven under pressure into the mosquito. Therefore, capillary feeding is faster than pool feeding^14^. In addition, the position of the mosquito positioning, particularly its legs, helps maintain the proboscis at a suitable height before penetration without excess pressure on its tip^15^. These studies did not directly address the pressure of the feeding pump, but they mentioned the existence of pressure inside the mosquito proboscis and its tip.

Next, the viscosity of the feeding solution may affect feeding behavior. The viscosities of the iodine solution and whole blood were measured using a rheometer with increasing shear rates in our previous study^8^. The iodine solution exhibits a similar viscosity range with that of blood, indicating a similar non-Newtonian fluid shear-thinning effect^8^. The feeding response to feeding solutions of different viscosities may be dissimilar for the two species of mosquitoes in this study. These distinctive differences between the two mosquito species may lead to different feeding behaviors. Further detailed studies are required to explain the effects of the distinctive differences on their behaviors.

In conclusion, the micro-3D reconstructed structures of the pumping organs of *An. sinensis* and *Ae. togoi* female mosquitoes were observed using SR-μCT. From the 3D reconstructed images of the pump systems, the cyclic variations in the pump volumes of the two mosquitoes were quantitatively compared. The blood-sucking pump organs and the feeding behaviors of the two species were distinct. These results will aid in understanding the mechanistic functions of the blood-feeding pump system as a disease vector.

## Methods

### Mosquito rearing and sample preparation

Following established rearing procedures^8^, mosquitoes (*Anopheles sinensis* s.l. and *Aedes togoi* Theobald 1907) were fed a 10% sugar solution at 27 °C with 80% humidity and a 16 h: 8 h light/dark cycle. Larvae were fed a slurry of ground fish food and baker’s yeast. After pupation, the mosquitoes were transferred to a cage and provided a 10% sucrose-soaked cotton rod. Among the piercing-sucking proboscises of a mosquito, the labrum, which has a feather-like opaque cuticle, does not pierce the skin of a host.

### Synchrotron X-ray microscopic computed tomography

All SR-μCT experiments were performed at the biomedical imaging (BMI) beamline of the Pohang Light Source-II (PLS-II) located in Pohang, Korea. This beamline was recently constructed and opened for general use in September 2013. A white beam emitted by the multi-pole wiggler source of the PLS-II 3 GeV storage ring was filtered by graphite with 1 mm thickness after passing through a double multilayer monochromator, resulting in photon energies of 10 KeV to 50 KeV. The optimal monochromatic X-ray energy for the present tomographic scanning experiments was experimentally determined to be approximately 24 KeV. The detector was positioned 20 downstream from the test sample for capturing phase-contrast images. The monochromatic X-ray image transmitted through the test sample was recorded by an imaging detector (Andor Zyla) with a spatial resolution of 2560 × 2160 pixels, upon which a YAG:Ce (30-μm thick) scintillation crystal was adhered. The X-ray image was converted on the scintillator into a visible image. All mosquito samples immersed in ethanol were placed at the tip of a heat-sealed pipette made of polypropylene that has a comparatively low X-ray absorption coefficient^16^. Parafilm was used to prevent alcohol evaporation. Each test sample was placed on a rotating sample stage, and 2D tomographic slice images of the sample were captured by rotating the stage from 0° to 180° in 0.5° intervals. The 3D morphological structure of each mosquito sample was reconstructed from the captured tomographic images using the Octopus image-processing program. To obtain the 3D volumetric image of the sample, a filtered back-projection algorithm was applied to the projection images. Cross-sectional images were stacked to produce a 3D structural image. The stacked images were rendered as a 3D morphological structure using the Amira® 5.3.3 image-analysis software (Visualization Sciences Group, Burlington, MA, USA).

### Scanning electron microscopy

Scanning electron microscopy (SEM) was employed to illustrate the morphological configurations of the test samples. Mosquito specimens were fixed with ethanol and submerged in hexamethyldisilazane or prepared by air-drying. The samples were then Ni-coated using a coater (Quorum Technology, SC7640 mode, East Sussex, United Kingdom) and examined in a Field Emission Scanning Electron Microscope (XL30S FEG, Philips Electron Optics B.V., the Netherlands) connected to an EDXS system at an acceleration voltage of 5 kV.

### Two-photon microscopy

The heads of the mosquitoes were dissected in PBS and fixed in 10% buffered formalin in a 0.1-M sodium phosphate buffer (pH 7.4) and 0.25% Triton X-100^17^. After fixation, the mosquito samples were incubated in phalloidin-FITC (Sigma-Aldrich, St. Louis, MO, USA) overnight at 4 °C. All fluorescent images were obtained using a Leica two-photon microscopy (TPM) system (TCS SP5II MP, Leica Microscopy Systems, GMBH) with a 20× objective lens (Leica Microscopy Systems, GMBH). The snapshot images captured by TPM were analyzed and processed with LAS AF 2.7 (Leica Microscopy Systems, GMBH).

### Statistical analysis

All data were statistically analyzed using the SPSS t-test (IBM, Chicago, IL, USA) at 95% coverage.

## Additional Information

**How to cite this article**: Ha, Y.-R. *et al.* Comparison of the functional features of the pump organs of *Anopheles sinensis* and *Aedes togoi*. *Sci. Rep.* **5**, 15148; doi: 10.1038/srep15148 (2015).

## Supplementary Material

Supplementary Information
