# Supplementary material for: Comparison of the functional features of the pump organs of Anopheles sinensis and Aedes togoi
Source: Sci Rep. 2015 Oct 14;5:15148. doi: 10.1038/srep15148 (PMC4604556; doi:10.1038/srep15148)
Supplement: Supplementary Information [file srep15148-s1.pdf]

Comparison of the functional features of the pump organs of *Anopheles sinensis* and *Aedes togoi*

Young-Ran Ha[1,2], Seung-Chul Lee[2,4], Seung-Jun Seo[3], Jeongeun Ryu [2,4], Dong-Kyu Lee[5], and Sang-Joon Lee[2,4]*

[1]Division of Integrative Bioscience and Bioengineering, Pohang University of Science and Technology, Pohang, 790-784, Republic of Korea

[2]Center for Biofluid and Biomimic Research, Pohang University of Science and Technology, Pohang, 790-784, Republic of Korea

[3]Pohang Accelerator Laboratory, Pohang University of Science and Technology, Pohang, 790-784, Republic of Korea

[4]Department of Mechanical Engineering, Pohang University of Science and Technology, Pohang, 790-784, Republic of Korea

[5]Department of Biology, Kosin University, Busan 516-36, Republic of Korea

Supplementary Methods

Two-photon microscopy

The midgut parts of the mosquitoes were dissected in PBS and fixed in 10% buffered formalin in 0.1 M sodium phosphate buffer (pH 7.4) and 0.25% Triton X-100 [17]. After fixation, the mosquito samples were incubated in phalloidin-FITC (Sigma–Aldrich, St. Louis, MO, USA) overnight at 4 °C. All fluorescent images were obtained by a Leica two-photon microscopy (TPM) system (TCS SP5II MP, Leica Microscopy Systems, GMBH) with a 20× objective lens (Leica Microscopy Systems, GMBH). The snapshot images captured by TPM

were analyzed and processed with LAS AF 2.7 software (Leica Microscopy Systems, GMBH).

Supplementary Figures

Figure S1.

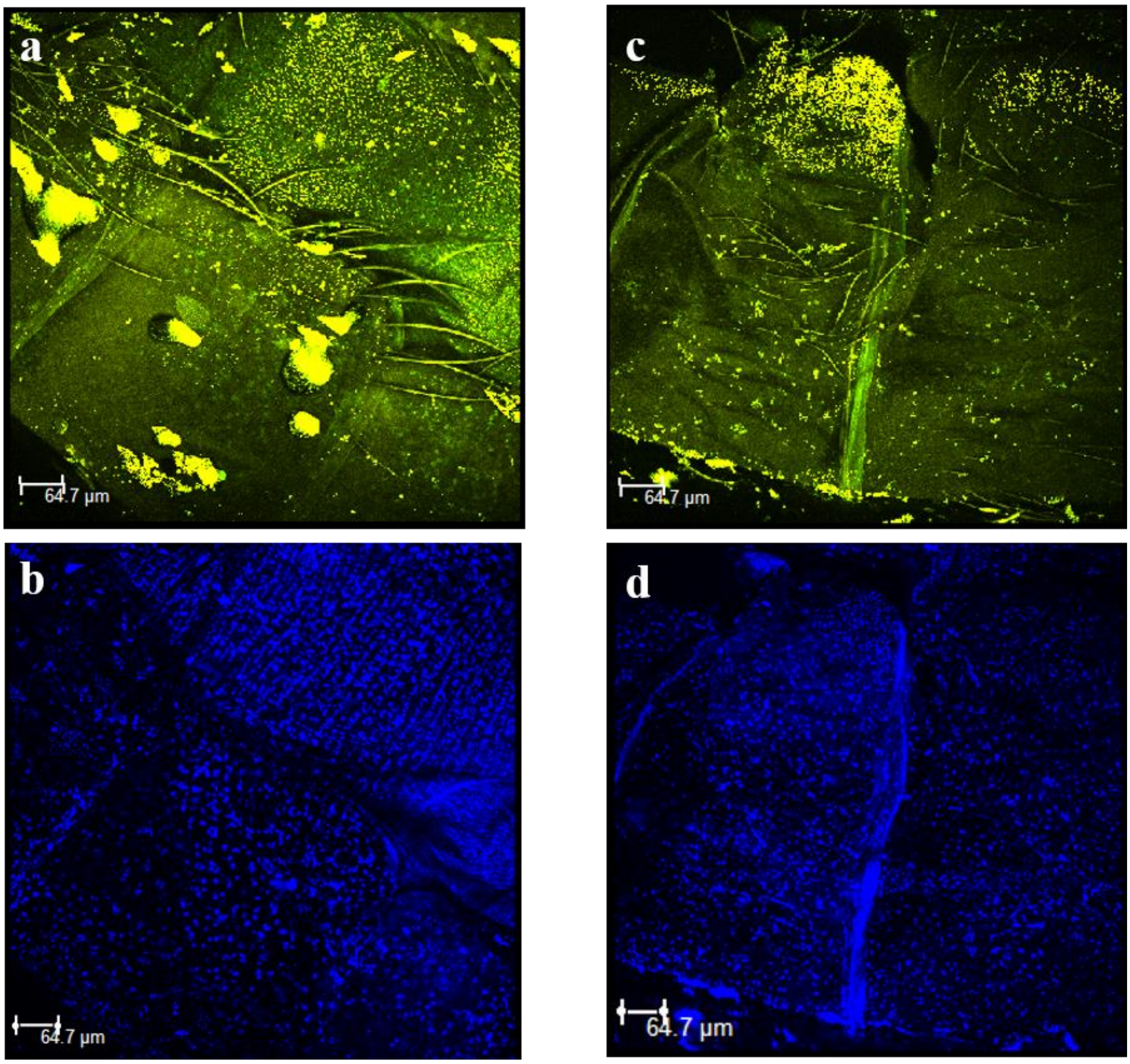

Supplementary figure legends

Figure S1. TPM images of the midgut of the two species of mosquitoes. Images of *Ae. togoi* stained with phalloidin-FITC (a) and Hoechst 33342 (b). Images of *An. sinensis* stained

with phalloidin-FITC (c) and Hoechst 33342 (d).
